# Depression in adolescents on treatment for rifampicin-susceptible TB in Lima, Peru

**DOI:** 10.5588/ijtldopen.25.0305

**Published:** 2025-11-12

**Authors:** V. Sanchez-Guzman, J.R. Tanzer, B. Roman-Sinche, J. Santacruz, C. Contreras, A. Desrosiers, L. Lecca, S.S. Chiang

**Affiliations:** 1 Department of Pediatrics, The Warren Alpert Medical School of Brown University, Providence, RI, USA;; 2 Biostatistics, Epidemiology, Research Design, and Informatics (BERDI) at Brown University Health, Providence, RI, USA;; 3 Socios En Salud, Lima, Peru;; 4 Department of Psychiatry and Human Behavior, The Warren Alpert Medical School of Brown University, Providence, RI, USA;; 5 Center for International Health Research, Rhode Island Hospital, Providence, RI, USA.

**Keywords:** tuberculosis, mental health, paediatric, symptoms, adverse events

## Abstract

**BACKGROUND:**

At least 1 million adolescents develop TB disease annually. Adolescents are also at risk for depression due to TB, but this is poorly understood.

**METHODS:**

This prospective cohort study aimed to identify the frequency and risk factors for depression amongst adolescents with rifampicin-susceptible TB in Lima, Peru. During weeks 3–5 of treatment, participants completed a survey that included socio-demographic characteristics, symptoms, treatment side effects, and the Patient Health Questionnaire-9 depression scale. Those with depression received psychological evaluation and were referred for treatment, with treatment intensity corresponding to depression severity. Applying k-means cluster analysis, we grouped participants by socio-demographic characteristics. We used generalised linear mixed-effects regression to model the relationship between cluster, symptoms and side effects, and depression.

**RESULTS:**

Of 249 participants, 98 (39%), 62 (25%), and 33 (13%) had mild, moderate, and severe depression, respectively. We identified three clusters; Cluster 1 – adolescents with lower social support and more prior trauma – had the highest frequency of depression. Across all clusters, symptoms and side effects correlated with depression.

**CONCLUSION:**

Given their high frequency of depression, adolescents on TB treatment – particularly those with trauma history, weak social support, or numerous symptoms and side effects – should be routinely screened for depression.

In 2023, 10.8 million people were diagnosed with TB, the world’s leading single-agent infectious killer.^1^ The high burden of depression in adults with TB has been widely reported, with an estimated pooled prevalence of 45.19%.^2^ Adolescence, defined as ages 10–19, is a developmental phase that entails multiple physical and psychosocial changes. Both the prevalence and incidence of TB rise in adolescence, with at least 1 million adolescents developing TB disease annually.^3^ Moreover, one in three adolescents experience depressive symptoms, as defined by the ‘Diagnostic and Statistical Manual of Mental Disorders’.^4,5^ Given the high prevalence of depression during adolescence and among people with TB, adolescents with TB are likely vulnerable to depression. Yet, few data have been published on this topic.^6^ We therefore aimed to identify the frequency and risk factors for depression among adolescents receiving treatment for rifampicin-susceptible TB. Stigma, inadequate social support, financial strain, substance use, severity and duration of disease, female gender, and past traumatic experiences have been identified as factors associated with depression both in adults with TB and healthy adolescents.^2,5,7–9^

We hypothesised that these variables would similarly be associated with depression in our cohort. Furthermore, physical symptoms – such as fatigue, pain, and weight loss – have been associated with depression among people with other medical conditions.^6,10,11^ Hence, we also investigated whether the combination of TB symptoms and treatment side effects – hereafter referred to as TB impact – was associated with depression in our cohort.

## METHODS

This prospective cohort study was conducted from 2020 to 2022 in Lima, Peru, in collaboration with *Socios En Salud* (SES; Peruvian branch of Partners in Health).^5^ Peru has a TB incidence of 173 per 100,000 population, the second highest in the Americas.^1^ In Peru, over half of the people with TB reside in Lima, the capital, and approximately one third of new diagnoses occur among people aged 15–24 years. TB treatment in Peru usually entails in-person directly observed therapy (DOT); home isolation for >2 months is often prescribed to adolescents.^5,12^

### Participant recruitment

Research staff visited participating public health centres throughout Lima and invited all consecutive, eligible patients with rifampicin-susceptible TB of any anatomic site to participate. Inclusion criteria were 1) age 10–19 years at TB treatment initiation; 2) availability of a parent or legal guardian to consent if the adolescent was <18 years old; 3) not yet initiated or have received <4 weeks of TB treatment. Adolescents with confirmed or suspected rifampicin-resistance were excluded.

### Data collection and referrals to mental health care

Each participant completed a self-administered survey on a tablet computer in a private space between weeks 3 and 5 of treatment using REDCap (Research Electronic Data Capture). The survey included socio-demographic information as well as the following validated scales: adverse childhood experiences (ACEs), alcohol use disorders identification test (AUDIT), an adolescent TB stigma scale, and the Patient Health Questionnaire-9 (PHQ-9) depression screening scale.^13–16^ The PHQ-9 consists of nine depressive symptoms; respondents receive points based on the frequency with which they have experienced each symptom in the prior 2 weeks: from 0 (‘not at all’) to 3 (‘nearly every day’). Points are added to generate a total score ranging from 0 to 27. Scores of 5–9 indicate mild depression; 10-14, moderate depression; and ≥15, severe depression. The PHQ-9 has been previously validated in Peru and other Latin American countries.^17^ Additionally, we assessed family support using a scale developed and previously published by our research team.^5,18^

**Figure 1.**
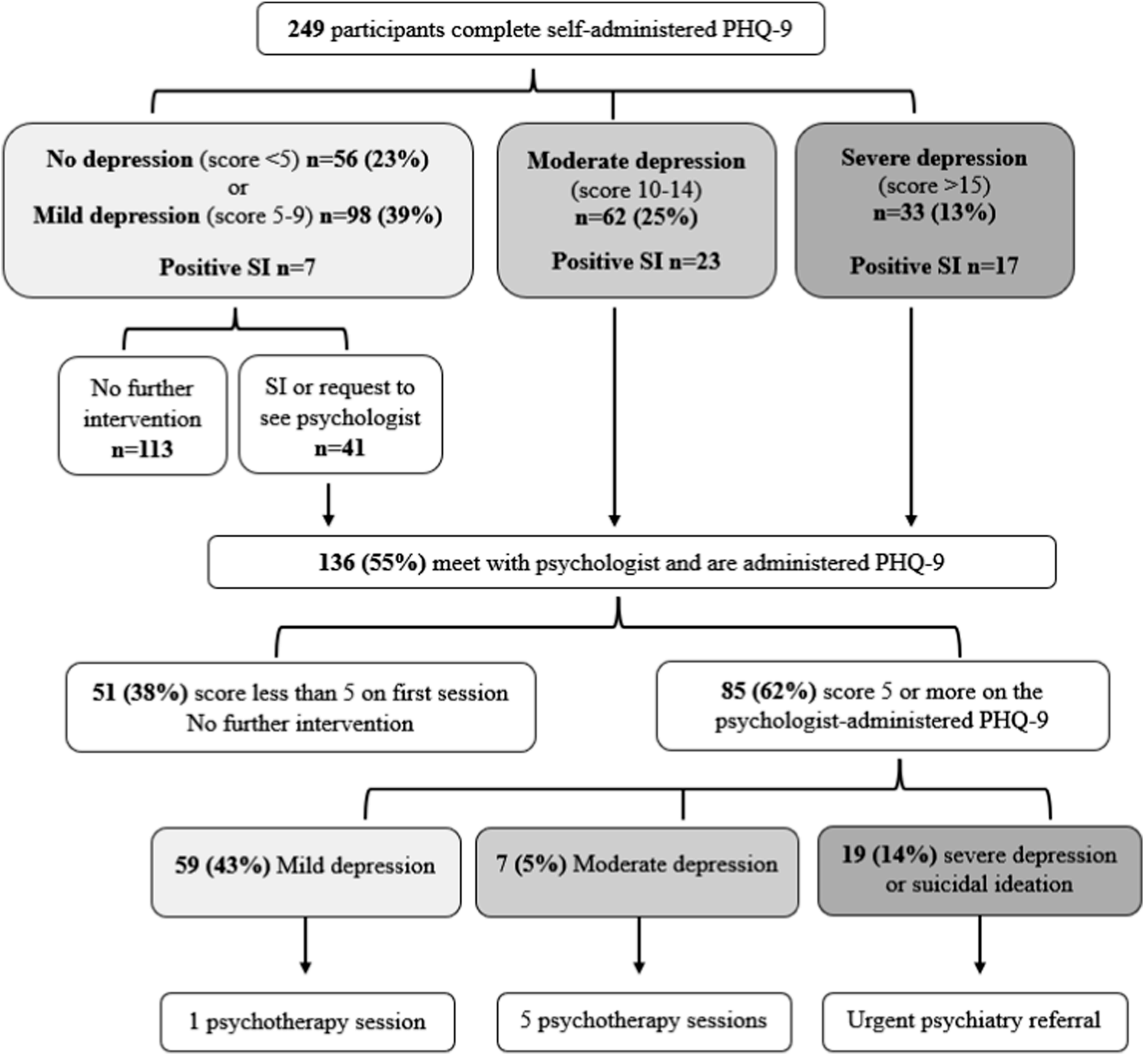
Flow chart of depression evaluations and referrals to care. Data collection and mental health referral algorithm. SI = suicidal ideation.

We applied a novel scale – TB impact – to summarise current TB symptoms and treatment side effects, given that it may be difficult for some adolescents to distinguish the two. The scale had 15 items with yes/no response options. It included nine symptoms – cough, fever, chills, fatigue, anorexia, night sweats, vomiting, weight loss, and haemoptysis – and six side effects – rash, vomiting, headache, nausea, abdominal pain, and fatigue. Participants received one point for each symptom and side effect they endorsed. Final scores were summed together, resulting in a value from 0 (no TB impact) to 15 (very high TB impact). Scores were then converted to z-scores. We also included single questions about food insecurity and illicit drug use. See Supplementary Data S2 for the questionnaire. A licensed psychologist employed by *Socios En Salud* provided evaluations within 24 h for participants with severe depression or suicidal ideation (included as a single question on the PHQ-9). Participants with moderate depression or those who did not meet these criteria but requested to meet with the psychologist were evaluated within 2 weeks. As part of the evaluation, the psychologist conducted a clinical interview, in which she probed for the causes of depression, and re-administered the PHQ-9, which determined the need for further treatment (Figure 1).

### Data analysis

All analyses were conducted using R (R Foundation for Statistical Computing, Vienna, Austria, 2024). Before conducting the main analysis, we used factor analysis and coefficient omega to confirm internal consistency reliability of all scales and comparison of factor loadings to confirm internal structure validity of the TB impact scale (Supplementary Data S1).

### Socio-demographic clusters

Given that many social characteristics hypothesised to be associated with depression were likely inter-related (e.g., traumatic experiences during childhood may lead to adolescent alcohol use), we applied k-means cluster analysis, using the *kamila* package, for data reduction. In this approach, participants were grouped based on numeric similarity across the following variables: age, gender, food insecurity, family support, TB-related stigma, AUDIT score, illicit drug use, and ACE score.^2,5,7,8^ We selected the number of clusters using an iterative process, examining inter-cluster descriptive statistics, until arriving at a clinically meaningful number.

### Definition of outcomes

We evaluated two measures of depression symptoms: 1) PHQ-9 score assessed on the self-administered survey, and 2) treatment intensity. Treatment intensity was based on the results of the evaluations, including the psychologist-administered PHQ-9 (Figure 1), and was scored as follows: 0.0 for no psychologist referral; 0.33 for psychological evaluation and one counselling session; 0.67 for psychological evaluation and five psychotherapy sessions; and 1.0 for psychological evaluation and urgent referral to acute psychiatric care. We included both outcomes because PHQ-9 scores have been reported to differ when self- versus psychologist-administered.^19^ The two approaches have their strengths and weaknesses: self-administration may elicit more honest answers due to depression-associated stigma, while administration by a psychologist may clarify misunderstandings about questionnaire items.

### Regression analysis

To evaluate the relationship between TB impact, clusters (socio-demographic characteristics), and depression, we used generalised linear mixed-effects regression. Depression was represented by the average of the self-administered PHQ-9 score and treatment intensity. Random effects were used to model the anticipated correlations between PHQ-9 scores and treatment intensity for each individual.

### Depression timing and underlying reasons

During the clinical interview, the psychologist asked and recorded the approximate onset date and reasons for depression on a standardised evaluation form with open-ended fields. We report these results descriptively for adolescents diagnosed with moderate or severe depression only because the information was not recorded for those with mild depression. Due to small numbers and the open-ended nature of these data fields, we did not perform hypothesis testing.

### Ethical statement

This study was approved by the institutional review boards of the Rhode Island Hospital and Peru’s National Institute of Health. Written informed consent was obtained from participants ≥18 years old or a parent/legal guardian of those <18 years old. Informed assents were obtained from minor participants.

## RESULTS

A total of 249 participants enrolled in the study and completed the study procedures. Participant characteristics can be found in Table 1. The adolescents in our study had a mean PHQ-9 score of 8.6, with a standard deviation of 4.7, and a median score of 8, with an interquartile range (IQR) of 5–11. There were 98 (39%), 62 (25%), and 33 (13%) adolescents who had mild, moderate, and severe depression, respectively. Across all depression categories, 47 (19%) participants had a positive screen for suicidal ideation. Of the 136 participants referred for psychological evaluation, 59 (43%), 7 (5%), and 19 (14%) adolescents had mild, moderate, and severe depression, respectively (Figure 1).

**Table 1.** Characteristics of socio-demographic clusters formed by k-means cluster analysis.

Traits	Mean (95% confidence interval)			
	All participants (n = 249)	Cluster 1 (n = 85)	Cluster 2 (n = 111)	Cluster 3 (n = 70)
Female (%)	36 (29, 42)	47 (36, 58)	60 (45, 74)	22 (15, 28)
Age (years)	16.56 (16.31, 16.84)	17.65 (17.33, 17.93)	12.95 (12.5, 13.43)	17.05 (16.76, 17.27)
Food insecurity^**A**^	1.99 (1.75, 2.05)	2.26 (2.01, 2.53)	1.90 (1.58, 2.27)	1.68 (1.46, 1.92)
Family support^**B**^	3.43 (3.36, 3.48)	3.08 (2.94, 3.19)	3.59 (3.51, 3.68)	3.59 (3.52, 3.66)
TB-related stigma^**C**^	2.3 (2.22, 2.39)	2.58 (2.44, 2.72)	2.44 (2.19, 2.7)	2.07 (1.96, 2.17)
AUDIT score**^D^**	0.68 (0.4, 1.02)	1.91 (1.07, 2.97)	0 (0, 0)	0.09 (0.03, 0.19)
Drug use (%)	14 (10, 18)	17 (10, 26)	0 (0, 0)	16 (10, 23)
ACE score^**E**^	2.02 (1.75, 2.26)	4.11 (3.69, 4.58)	0.98 (0.62, 1.48)	0.97 (0.81, 1.11)

A Food insecurity assessed with single question scored on a five-point scale.

B Family support scale range 1–5; scored from 15 items (omega reliability = 0.75).

C TB-related stigma scale adapted for adolescents: score range 10–50 (omega reliability = 0.85). The scale consists of 12 questions scored on a 5-point Likert scale ranging from 1 (*never*) to 5 (*always*). Participants report the degree to which they experience the different aspects of stigma. This scale has been previously validated by our research team.^5^

D AUDIT = alcohol use disorders identification test; score range 0–40. The AUDIT scale consists of 10 items and has been previously validated for assessment of alcohol use disorder.

E ACE = adverse childhood experiences; score range 0–10 (reliability unable to be assessed because responses to individual items are not recorded). The ACE scale is composed of 10 experiences. The participant indicates the number of experiences they have had, and the total is their score.

Based on the results of the k-means cluster analysis, we grouped participants into three socio-demographic clusters (Table 1). Cluster 1 consisted of adolescents who were older (mean: 17.65 years; 95% CI: 17.33–17.93 years) and had the least family support (i.e., lowest average family support score), most prior trauma (i.e., highest average ACE score), and most alcohol use (i.e., highest average AUDIT score). Cluster 2 consisted of distinctly younger participants (mean: 12.95 years; 95% CI: 12.50–13.43 years) with high endorsement of family support and minimal drug or alcohol use. Cluster 3 had a similar age range as Cluster 1 (mean: 17.05; 95% CI: 16.76–17.27) but had the highest proportion of male adolescents, low ACE scores, and high endorsement of family support.

**Figure 2.**
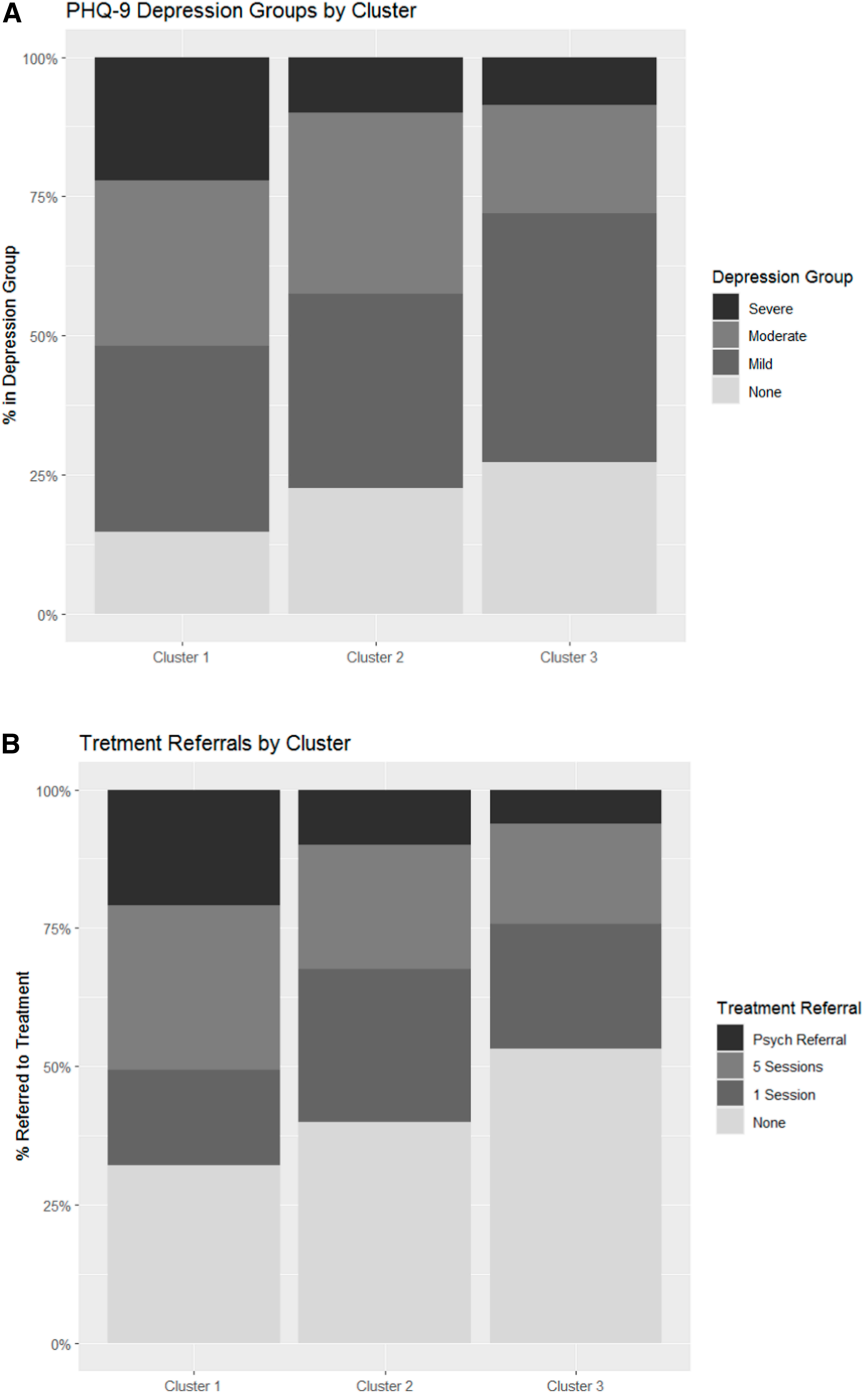
Self-administered PHQ-9 scores and treatment needs by cluster. **A:** The percentage of participants in each socio-demographic cluster with none, mild, moderate, and severe depression as determined by the self-administered PHQ-9 scores. **B:** The same clusters, now indicating the percentages of treatment referral types (i.e., treatment intensity), as determined by the self-administered and psychologist-administered PHQ-9 scales. PHQ-9 = Patient Health Questionnaire-9.

**Figure 3.**
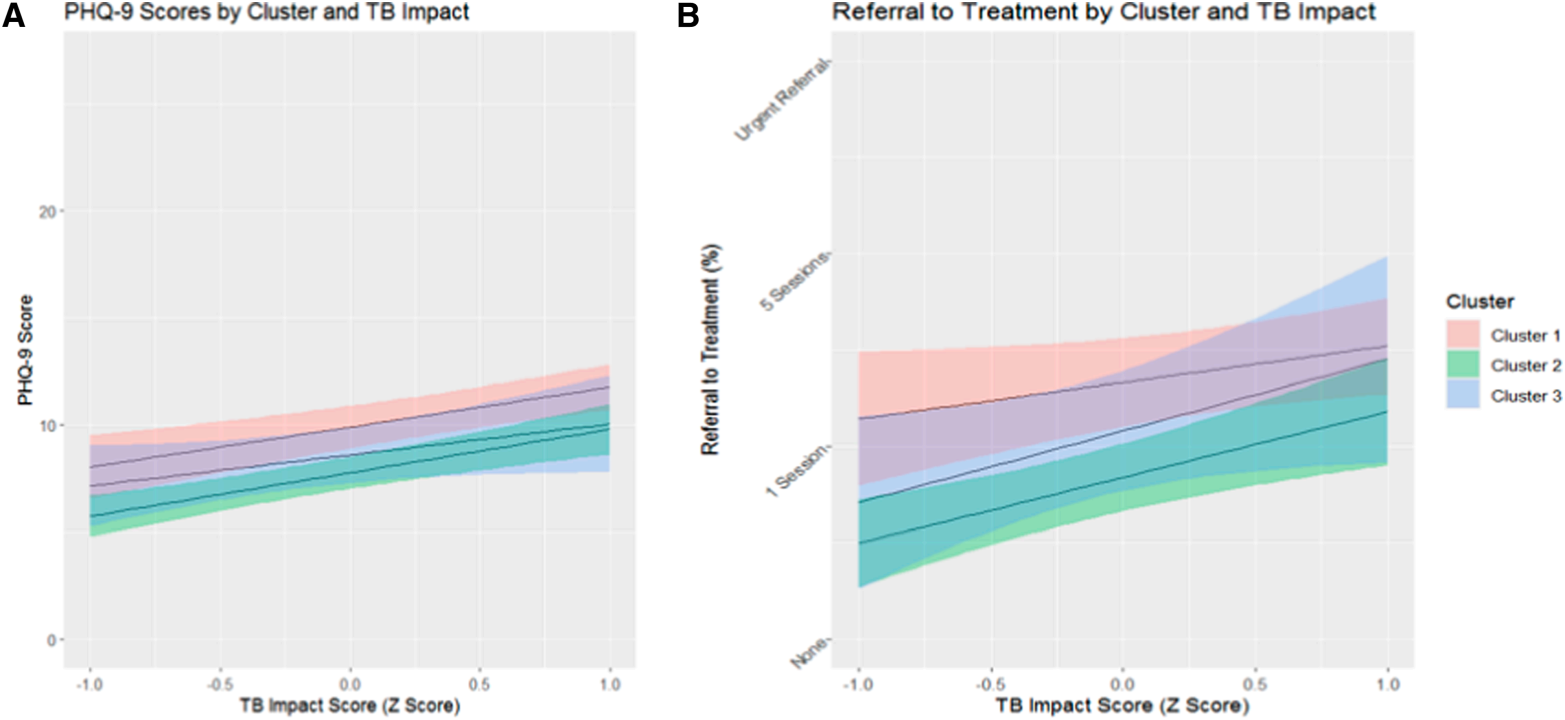
**A:** The relationship between PHQ-9 scores and TB impact scores (which represent a combination of TB symptoms and treatment side effects) for each cluster. **B:** The relationship between TB impact scores and treatment intensity. PHQ-9 = Patient Health Questionnaire-9.

Self-administered PHQ-9 scores and treatment intensity had a correlation of 0.65. Cluster 1 had the highest frequencies of severe depression and urgent referrals to psychiatric care (i.e., greatest treatment intensity). Cluster 3 had the least number of participants with severe depression (Figure 2). In all clusters, adolescents with higher TB impact scores reported more severe depression on the self-administered PHQ-9 (*t*(485) = 5.35, *P* < 0.0001; Figure 3A).

Adolescents across all clusters who had higher TB impact scores also were more likely to have greater treatment needs (*t*(485) = 3.78, *P* = 0.0002; Figure 3B). (Psychometric properties of the TB impact scale were acceptable and are detailed in Supplementary Data Tables S1 and S2.)

Over 50% of adolescents with severe depression reported that their depressive symptoms began before TB treatment, and factors other than TB contributed to depression in three quarters of these participants. In contrast, most adolescents with moderate depression reported that their depression began around the time of TB treatment and was due solely to TB (Table 2).

**Table 2.** Onset and causes of depression, as reported by adolescents during clinical interviews with the psychologist.

	Depression severity, as diagnosed by the psychologist-administered PHQ-9^**A**^	
	Severe depression, PHQ-9 score ≥15 (n = 19)	Moderate depression, PHQ-9 score 10–14 (n = 7)
Onset of depression		
• Around the same time or after TB treatment	6 (31.56)	6 (85.71)
• >1 year before TB treatment started	5 (26.32)	0 (0)
• <1 year before TB treatment	7 (36.84)	1 (14.29)
• Unsure or did not want to say	1 (5.26)	0 (0)
Cause of depression		
• TB-related only	1 (5.26)	4 (57.14)
• Other factors only^**B**^	9 (47.37)	2 (28.57)
• TB-related and other factors	6 (31.58)	1 (14.29)
• Unsure or did not want to say	3 (15.79)	0 (0)

A Results were not recorded for adolescents with mild depression.

B The most commonly reported other factors were related to family conflict and/or socio-economic difficulties.

PHQ-9 = Patient Health Questionnaire-9.

## DISCUSSION

In this study, we sought to understand the frequency, severity, and predictors of depression among adolescents with TB in Lima. Findings suggest a high frequency of depression symptoms, and those experiencing worse impact of TB had more severe depression with greater treatment needs. The high proportion of depression among our study participants is consistent with prior research on depression among adolescents with TB in other contexts. A recent study in Romania reported that 146 out of 158 (92.4%) children and adolescents 7–18 years old receiving TB treatment demonstrated clinically significant depressive symptoms, as assessed by the Children’s Depression Inventory, and those aged 15–18 were more likely to report greater depression severity.^6^ Among adolescents with TB in Cape Town, South Africa, PHQ-9 scores (median: 13; IQR: 11–17) were comparable to those found in our cohort.^18^ Our results underscore the importance of routine depression screening and mental health services for adolescents living with TB in Lima. This conclusion is strengthened by evidence from qualitative studies from Lima, Cape Town, and Gaborone, Botswana, in which adolescents with TB, their caregivers, and TB treatment providers strongly endorsed the need for more mental health services for adolescents with TB.^20–22^

Our findings also indicated that adolescents with TB who experienced greater severity of TB symptoms and side effects reported higher levels of depression; this pattern was consistent across socio-demographic clusters. It is possible that adolescents become more depressed because they have more physical discomfort due to TB, but it is also possible that adolescents with higher depression symptoms experienced more difficulty tolerating TB-related physical discomfort. Future studies using longitudinal designs that include biobehavioural measures (i.e., passive sensing data and physiological measures) could help understand directionality. Overall, findings suggest that adolescents with greater degrees of physical comfort may be more vulnerable to depression and should be targeted for depression screening. Additionally, treating TB side effects or symptoms (i.e., medication for nausea) may help mitigate depression symptoms.

Results from the clinical interviews showed that severe depression in our cohort often predated TB treatment and was related to factors other than TB. However, moderate depression was mostly attributed to TB, which is consistent with the reported onset concomitant with or after TB treatment initiation. Findings are consistent with the statistical associations that we observed between adverse socio-demographic circumstances (food insecurity, higher ACE scores, lower family support) and depression severity, as well as with prior research supporting associations between poor mental health among adolescents and childhood trauma, low social support, and suboptimal parenting.^14,23,24^

Scarce mental health care resources in many TB-endemic settings are an important limitation to overcome. However, various lost-cost approaches have been used effectively. In Lima during the COVID-19 pandemic, *Socios En Salud* staff used chatbots to screen adults on TB treatment for depression and provided virtual counselling and psychotherapy sessions for those with mild and moderate depression, and urgent psychiatric referral for those with severe depression (similar to the referral procedures implemented in our study).^25^ Re-evaluation of these participants 6 months later showed significant reductions in PHQ-9 scores. This low-intensity intervention may be extended to adolescents in Lima and other similar settings. Other examples of low-intensity interventions include peer-led psycho-education and social support groups for adolescents and their caregivers.^26,27^ Culturally adapting existing evidence-based mental health interventions tailored for youth living with chronic diseases may be one strategy to address mental health needs of Peruvian youth living with TB, as well as exploring the integration of evidence-based mental health services within TB care settings.^28^ Future research to evaluate mental health interventions among adolescents with TB should include economic analyses to compare the costs of the interventions being assessed against quantified societal costs associated with untreated depression among adolescents; these data would inform policy and investments in mental health services.

Our study had several limitations. Adolescents who did not have a parent or guardian available to sign informed consent were excluded, which may have skewed the sample towards participants with more family support. Adolescents with TB who had a strain with suspected or confirmed resistance to rifampicin were also excluded; this population may have experienced additional medical and social challenges. Finally, we did not assess anxiety or traumatic stress, which, together with depression, provides a more comprehensive understanding of an adolescent’s mental health. Future studies should measure these disorders in adolescents with TB to better inform interventions. Other future research directions include participant follow-up to assess depression symptom trajectory of adolescents with TB over time and in response to mental health interventions.

## CONCLUSION

Given their high rates of depression, adolescents on TB treatment – particularly those with trauma history, weak social support, or numerous symptoms and side effects – should be routinely screened for depression. There is an urgent need to develop, pilot, and evaluate the efficacy of interventions to provide mental health care for adolescents with TB in resource-limited settings.

## Supplementary Material


