# Supplementary material for: Depression in adolescents on treatment for rifampicin-susceptible TB in Lima, Peru
Source: IJTLD Open. 2025 Nov 12;2(11):639–45. doi: 10.5588/ijtldopen.25.0305 (PMC12617082; doi:10.5588/ijtldopen.25.0305)
Supplement: Supplementary file 1 [file ijtldopen25-0305_supplementarydata1.pdf]

SUPPLEMENTARY TEXT 1: PSYCHOMETRIC ANALYSIS

Validity analysis was performed for questionnaires included in the analysis and the tuberculosis (TB) impact score, to assess reliability and validity. Reliability is a mathematical calculation to evaluate the likelihood that two scores provided to similar individuals will be the same. Possible values for reliability range from 0.00 to 1.00, with reliability >0.70 often considered acceptable. Validity, on the other hand, represents the extent to which the questionnaire can be interpreted as representing the trait it purports to measure. While empirical estimates often inform assessments of validity, there are no inherent standards for what is acceptable. It is often guided by evaluation of questionnaire responses to evaluate if it acts the way it is anticipated to.

To estimate reliability, internal consistency reliability was estimated by McDonald's Omega, which is based on the factor loadings. Mathematically calculated from the correlation matrix, factor loadings represent the extent to which each individual question is correlated with all other questions. The factor loadings summarize these values across all pairs of questions by eigenvalue decomposition. This simplifies interpretation and reduces the risk of spurious correlation because rather than looking at all pairwise correlations, which increase exponentially as questions are added to the questionnaire, a single loading value can be examined for each question. Coefficient omega reliability is based on the magnitude of the loadings. Larger loadings imply greater associations across all questions, and result in greater implied reliability. Omega reliability was estimated for all multi-item questionnaires, except for the adverse childhood experiences (ACE) questionnaire, for which itemized responses were not available.

To assess the internal structure validity of the new TB impact score, we examined the values of individual factor loadings to assess internal structure validity. The values of the loadings indicate which questions contributed more meaningful variation to the final scores, and which questions were more unique. The interpretation of what each scale represents is based on which loadings are highest. As an example, consider a flu symptomatology questionnaire. A runny nose is not a specific symptom, as it could be associated with a cold, allergies, or something else entirely. Comparatively, body aches are more specific to the flu, making it a more useful symptom to assess when determining whether someone may have the flu.

Table S1 presents the results of the psychometric analysis of the TB impact scale. One point was given for each symptom or side effect that was experienced. Final scores were summed together, resulting in a value from 0 (no TB impact) to 15 (very high TB impact). Reliability was acceptable (omega = 0.72, 95% CI: 0.66, 0.77). In terms of internal structure validity, the most meaningful items were fatigue, vomiting, and nausea. In terms of the distribution of scores, the average was low (mean = 2.76) relative to the variation in scores (SD = 2.10); however, there was no excessive skew (0.78) or kurtosis (0.11; see Table S2).

Table S2 reports the univariate distribution of all scales used in this study. It was not possible to estimate reliability for ACE scores, as the itemized scores were not available during data collection. The family support, social stigma, and PHQ-9 scales indicated good reliability (omega = 0.75, 0.85, and 0.84 respectively). Additionally, most

scales indicated appropriate skew and kurtosis (i.e., < |1.0|). The exception was the AUDIT, for which scores were on average very low (mean = 0.68, possible scores from 0-40) with very large skew (5.94) and kurtosis (41.58). This is likely reflective of the infrequency of alcohol use in our study population.

TABLE S1: TB IMPACT SCORE

Experience	Percent Endorsement	Loading
Symptoms		
Cough	43%	0.20
Fever	2%	0.24
Chills	4%	0.16
Fatigue	45%	0.50
Anorexia	15%	0.56
Night Sweats	12%	0.40
Vomiting	6%	0.47
Weight Loss	8%	0.38
Hemoptysis	2%	0.23
Treatment Side Effects		
Rash	9%	0.22
Vomiting	12%	0.51
Headache	25%	0.40
Nausea	25%	0.56
Abdominal Pain	22%	0.34
Fatigue	44%	0.51
Internal Consistency Reliability:	0.72, 95% CI [0.66, 0.77]	

Note: If the symptom or side effect was endorsed, it was scored as 1; otherwise, it was scored as 0. A summed score was calculated, with possible scores ranging from 0 to 15. In the regression analysis, scores were incorporated as z-scores because we did not have data to validate the reported symptoms and side effects, so we did not want to imply more precision than we had evidence of. Higher scores reasonably can be interpreted as representing worse experience with TB symptoms and treatment side effects.

TABLE S2: SAMPLE CHARACTERISTICS OF ALL SCALES

Scale	Items	Mean	SD	Skew	Kurtosis	Reliability
Family Support (1-5)	15	3.43	0.52	-0.88	0.96	0.75
Social Stigma (1-5)	12	2.30	0.70	0.63	0.15	0.85
AUDIT Score (0-40)	10	0.68	2.71	5.94	41.58	*
ACE Score (0-10)	10	2.02	2.02	0.98	0.06	*
PHQ-9 (0-27)	9	0.81	0.45	0.65	0.13	0.84
TB Impact Score (0-15)	15	2.76	2.10	0.78	0.11	0.72
Criterion	More is better	Not too close to upper or lower limit	More is better	< |1.0|	< |1.0|	> 0.70

*Itemized scores not available; thus, reliability could not be estimated

TABLE S3: REGRESSION RESULTS

Coefficient	Estimate	SE	$t(485)$	p
Intercept	0.367	0.019	19.57	< 0.0001
<u>Main Effects</u>				
TB Impact	0.069	0.016	4.45	< 0.0001
Group 3	-0.079	0.023	-3.51	0.0005
Group 2	-0.048	0.030	-1.59	0.1129
Referral Outcome	0.077	0.031	2.51	0.0124
<u>Interactions</u>				
TB Impact * Group 2	-0.015	0.034	-0.45	0.6558
TB Impact * Group 3	0.006	0.021	0.29	0.7704
TB Impact * Referral Outcome	-0.006	0.027	-0.23	0.8205
Group 2 * Referral Outcome	-0.035	0.052	-0.68	0.4994
Group 3 * Referral Outcome	-0.085	0.039	-2.20	0.0286
TB Impact * Group 2 * Referral Outcome	0.077	0.059	1.31	0.1908
TB Impact * Group 3 * Referral Outcome	0.045	0.037	1.22	0.2231

SUPPLEMENTARY TEXT 2: QUESTIONNAIRE

We would like to invite you to participate in this survey about tuberculosis treatment for adolescents. If you do not understand a word or would like us to clarify a question, please let us know. In this survey there are questions that might involve some personal topics. I want to remind you that your participation in this survey is completely voluntary. Additionally, all the information that you share with us on this survey will be monitored in a strictly confidential manner (unless you state that you are going to hurt yourself or others). This means that we will not share your answers with your medical providers or your family. All of the information will remain anonymous and be shared only with members of our study team.

Household Information

Please tell us your gender:

- Male
- Female
- I was born male but I feel female
- I was born female but I feel male

How old are you?

- 10 years old
- 11 years old
- 12 years old

- 13 years old
- 14 years old
- 15 years old
- 16 years old
- 17 years old
- 18 years old
- 19 years old

For the next question, please respond with ONE of the following options: Always, Often, Sometimes, Rarely, or Never.

Knowing that it is necessary to eat 3 times per day for a healthy diet, how often have you had to eat less on per day because there was not enough money in the house to buy food?

Always - Often - Sometimes - Rarely - Never

TB Symptoms

What symptoms do you *currently* have? (select ALL that apply)

- Cough
- Fever
- Chills (intense cold sensation followed by slight tremors of the body)
- Fatigue
- Loss of Appetite

- Sweating at night
- Vomiting
- Weight loss
- Hemoptysis (losing blood)
- I did not have symptoms
- Other; please specify: ______________________________

What side effects or discomfort do you have or have you had because of the medications? (select ALL that apply)

- Rash
- Vomiting
- Headache
- Nausea
- Abdominal pain
- Fatigue
- No discomfort
- Others; please specify: ____________________________

How often do you experience discomfort as a result of taking your TB medications?

Always - Often - Sometimes - Rarely - Never

Family Relationships

In the following questions, please respond by selecting ONE of the following options: Always, Often, Sometimes, Rarely, Never.

My mother/father/guardian treats me with kindness.

Always - Often - Sometimes - Rarely - Never

I get along well with my mother/father/guardian.

Always - Often - Sometimes - Rarely - Never

I get along well with other family members.

Always - Often - Sometimes - Rarely - Never

I confide in my family.

Always - Often - Sometimes - Rarely - Never

I have secrets that I do not share with my mother/father/guardian.

Always - Often - Sometimes - Rarely - Never

My mother/father/guardian supports me emotionally with my TB treatment.

Always - Often - Sometimes - Rarely - Never

Other family members support me emotionally with my TB treatment.

Always - Often - Sometimes - Rarely - Never

My friends support me emotionally with my TB treatment.

Always - Often - Sometimes - Rarely - Never

Generally, my mother/father/guardian and I always have had emotional support from

other family members.

Always - Often - Sometimes - Rarely - Never

Someone from home accompanies me when I go to the health center for my medications.

Always - Often - Sometimes - Rarely - Never

My other family members (outside of my immediate family, in other words people other than parents or siblings) know that I have TB.

Always - Often - Sometimes - Rarely - Never

My mother/father/guardian does not like my friends.

Always - Often - Sometimes - Rarely - Never

I fight with my mother/father/guardian.

Always - Often - Sometimes - Rarely - Never

My TB illness cases my parents and family members to argue.

Always - Often - Sometimes - Rarely - Never

Generally, I am happy with my relationship with my mother/father/guardian.

Always - Often - Sometimes - Rarely - Never

How do I feel about having TB?

I am afraid to tell my family members that I have TB.

Always - Often - Sometimes - Rarely - Never

I am afraid to go to the health center to get my pills because I am afraid others will see me.

Always - Often - Sometimes - Rarely - Never

I am afraid to tell people that I have TB because they might think that I also have AIDS.

Always - Often - Sometimes - Rarely - Never

I feel guilty because I think I got sick with TB because I did not follow a healthy diet.

Always - Often - Sometimes - Rarely - Never

I feel guilty because I think I got sick with TB because of smoking, drinking alcohol, or using other drugs.

Always - Often - Sometimes - Rarely - Never

I feel guilty about getting TB because I am a burden for my family.

Always - Often - Sometimes - Rarely - Never

I am careful when choosing whom to speak to about my TB.

Always - Often - Sometimes - Rarely - Never

I might lose my friends if I tell them I have TB.

Always - Often - Sometimes - Rarely - Never

I am afraid to tell people who are not family members that I have TB.

Always - Often - Sometimes - Rarely - Never

I feel alone because of my TB.

Always - Often - Sometimes - Rarely - Never

I feel upset by the way people react when they find out that I have TB.

Always - Often - Sometimes - Rarely - Never

I keep my distance from others to avoid passing on my TB to them.

Always - Often - Sometimes - Rarely - Never

Mood and emotions

The following questions are about your feelings and emotions. How often have you felt each of the following symptoms during the PAST TWO WEEKS? For every symptom, select the option that best describes how you have felt.

Have you ever felt down, depressed, irritable, or hopeless?

Never - Some days - More than half the days - Almost every day

Have you felt little interest or pleasure in doing things?

Never - Some days - More than half the days - Almost every day

Have you had problems falling asleep or staying asleep?

Never - Some days - More than half the days - Almost every day

Or by contrast, have you been sleeping too much?

Never - Some days - More than half the days - Almost every day

Have you ever felt tired or with little energy?

Never - Some days - More than half the days - Almost every day

Have you had a lowered appetite or have you lost weight?

Never - Some days - More than half the days - Almost every day

Or, by contrast, have you eaten excessively?

Never - Some days - More than half the days - Almost every day

Have you felt bad about yourself or have you thought of yourself as a failure, or someone who has disappointed yourself or your family?

Never - Some days - More than half the days - Almost every day

Have you had difficulties concentrating in certain activities, like school work, reading, or watching television?

Never - Some days - More than half the days - Almost every day

Have you ever moved or talked so slowly that other people have taken notice?

Never - Some days - More than half the days - Almost every day

Or, by contrast, have you been so restless that you haven't been able to stop moving more than usual?

Never - Some days - More than half the days - Almost every day

Have you ever thought that it would be better to be dead or have you thought of hurting yourself in some way?

Never - Some days - More than half the days - Almost every day

Alcohol consumption

It is important that we ask you certain questions about your consumption of alcohol. Your responses will be confidential, so please be honest. In the past 12 months:

How often do you drink alcoholic beverages?

- Never
- Once or less per month
- From 2-4 times per month
- From 2-3 times per week
- 4 or more times per week

How many alcoholic drinks do you normally have on a typical day?

- 1 to 2
- 3 to 4
- 5 to 6
- 7 to 9
- 10 or more

How often do you have 6 or more alcoholic beverages in only one day?

Never - Monthly - Weekly - Almost daily - Daily

How often in the past year have you been unable to stop drinking once you started?

Never - Monthly - Weekly - Almost daily - Daily

How often in the past year have you not been able to do what was expected of you because you drank?

Never - Monthly - Weekly - Almost daily - Daily

How often in the past year have you had to drink alcohol in order to recover after drinking too much the previous day (or to avoid a hangover)?

Never - Monthly - Weekly - Almost daily - Daily

How often in the past year have you felt remorse or guilt after drinking?

Never - Monthly - Weekly - Almost daily - Daily

How often in the past year have you been unable to remember what happened the night before because you had been drinking?

Never - Monthly - Weekly - Almost daily - Daily

Have you or another person gotten injured because you had been drinking?

- No
- Yes, but not in the past year
- Yes, in the past year

Has someone you know (friend, doctor, or professional) been concerned about your

consumption of alcohol or has suggested that you stop drinking?

- No
- Yes, but not in the past year
- Yes, in the past year

Drug use

At least once have you taken any of these drugs (without medical prescription)?

PLEASE MARK ALL THAT YOU HAVE TAKEN AND HOW OFTEN. One of the following options will be selected for the items below:

- Never
- 1-2 times
- Every month
- Every week
- Daily or almost daily

a. Tobacco (cigarettes, pipes, cigars, chewing tobacco, pipe, etc.)

b. Cannabis (marijuana, hashish, etc.)

c. Cocaine (coca, crack, etc.)

d. Amphetamines or other stimulants (speed, ecstasy, ice, Ritalin, Dexedrine, slimming pills, methamphetamine, etc.)

e. Inhalants (glues, terocal, gasoline / naphtha, glue, solvents, Poppers etc.)

f. Tranquilizers or sleeping pills (valium / diazepam, Trankimazin / Alprazolam / Xanax, Orfidal / Lorazepam, Rohipnol, Librium, Ativan, GHB, Nembutal, Seconal, Phenobarbital, etc.)

g. Hallucinogens (LSD, acids, ketamine, mezcalin, PCP, angel powder, etc.)

h. Opioids (heroin, methadone, codeine, morphine, dolantine / pethidine, OxyContin, Darvon, Vicodin, Dilaudid, Demerol, Lomotil, Percodan, etc.)

Others; please specify: ___________________________

In the past 12 Months - For the questions below, one of the following will be selected:

- No
- Yes
- Prefer not to answer

Have you felt the need or desire to take the drugs you mentioned that you were unable to resist?

Have you tried to not take or have you had difficulties with stopping before becoming intoxicated?

When you take less drugs or do not take them at all, do you feel symptoms from withdrawal? (pain, shaking, fever, weak feelings, diarrhea, nausea, sweating, elevated heartbeat, difficulty sleeping, feeling agitated, irritable, or depressed? Or, have you taken another substance to avoid feeling these withdrawal symptoms?

Have you noticed that you have to take a higher dose of drugs to get the same effects has before?

Have you done less activities (in your free time, at rest, or daily activities) because you were taking drugs?

Have you continued taking the drugs you mentioned even though you know they result in physical and psychological problems?

In the past 12 months - For the questions below, one of the following will be selected:

- No
- Yes
- Prefer not to answer

Since you last took the drugs you mentioned, have you had health problems like accidental overdose, chronic cough, convulsions, infections, problems with your liver, or an injury?

Since you started taking drugs, have you had psychological problems, like not being interested in anything, feeling sad, not trusting other people, having thoughts of being persecuted or other strange thoughts?

Have you had problems at school, at work, or at home because of taking drugs?

Adverse Childhood Experiences

Many children and adolescents have stressful experiences that may impact their health and well-being. Read the following statements. Tell us which ones which may apply to you by writing the total number in the corresponding box. Do NOT mark nor indicate the specific statements that apply to you.

Of the following statements, HOW MANY are applicable to you? _____ (0 – 10 points)

At some point in time since you were born:

- Your parents or guardians divorced or separated.
- You lived with someone that was in jail or prison.
- You lived with someone that suffered from depression, had a mental illness, or attempted suicide.
- You saw or heard people you trust hurt themselves or threaten to hurt themselves.
- A person that you trust has been rude to you, insulted you, humiliated you, or criticized you in a way that scared you or made you afraid that you would be harmed in a physical manner.
- Someone touched you in your private parts or asked you to touch their private parts in a sexual manner that you did not want, against your will, or that made you feel uncomfortable.
- You have lacked food, clothing, a place to live or someone to protect you.
- Someone pushed, grabbed, slapped, threw something or hit you with enough force that it left a mark or bruise.
- You lived with someone that had an alcoholic or drug problem.
- You have felt that no one is there to support, love, or protect you.

If you would like to talk more with the authorities of the study about any of these problems, and/or you would like that they contact you to help you find help or support, mark “Yes”
